# Clinical and Biochemical Characteristics of Severe Hypothyroidism Due to Autoimmune Thyroiditis in Children

**DOI:** 10.3389/fendo.2020.00364

**Published:** 2020-07-08

**Authors:** Anna Małgorzata Kucharska, Ewelina Witkowska-Sȩdek, Dominika Labochka, Małgorzata Rumińska

**Affiliations:** Department of Paediatrics and Endocrinology, Medical University of Warsaw, Warsaw, Poland

**Keywords:** severe autoimmune hypothyroidism, symptoms, hypertrichosis, pituitary hypertrophy, children

## Abstract

**Introduction:** In the majority of countries, autoimmune thyroiditis is the main cause of acquired hypothyroidism in children. Typically, the natural course of the disease is initially insidious and the diagnosis is incidental. There are some children who develop severe hypothyroidism without a proper diagnosis. The aim of the study was to analyze the clinical and biochemical profiles of children with severe primary hypothyroidism due to autoimmune thyroiditis.

**Materials and Methods:** We analyzed the records of 354 patients diagnosed between 2009 and 2019 with autoimmune thyroiditis. Only patients with TSH above 100 μIU/mL, associated with decreased free thyroxine and the presence of antithyroid antibodies, were enrolled in the study. The analysis encompassed clinical symptoms, thyroid and biochemical status, bone age, and imaging.

**Results:** Twenty-six children were enrolled in the study. The mean age at diagnosis was 10.26 ± 3.3 years, with a female preponderance of 1.8:1. The most frequent symptom was growth impairment (77%) and weight gain (58%). Goiters were present in 42% of patients. Less common findings were pituitary hypertrophy (four patients) and hypertrichosis (three patients). Median values at the time of diagnosis were TSH 454.3 uIU/ml (295.0–879.4), anti-TPO antibodies 1,090 IU/ml, and anti-Tg antibodies 195 IU/ml. Anti-TSHR ab were evaluated only in six out of the 26 patients. The characteristic biochemical profile was correlated with the grade of hypothyroidism, and the strongest correlations were found with CBC parameters, lipid profile, aminotransferases, and creatine.

**Conclusion:** In children with severe hypothyroidism, the most sensitive symptoms are growth arrest and weight gain despite the fact that, in some children, the auxological parameters at presentation could be within normal values for the population. The specific biochemical profile closely correlates to the severity of thyroid hormone deficiency and involves mostly erythropoiesis, liver function, and kidney function. Pituitary enlargement should be considered in each child with severe hypothyroidism. It is necessary to conduct prospective studies evaluating the actual frequency of anti-TSHR antibodies and pituitary enlargement in children with extremely high TSH, especially those presenting without goiters.

## Introduction

Autoimmune thyroiditis (AIT) is the main cause of acquired hypothyroidism in children (1, 2). Typically, the natural course of disease is initially insidious and many patients are diagnosed incidentally before they present overt hypothyroidism (3). There are also some children who develop severe hypothyroidism for several months without a proper diagnosis.

The most common form of AIT is classic Hashimoto's disease with goiter—a high level of antithyroid antibodies and infiltration of the thyroid gland by macrophages and lymphocytes that form the specific thyroid lymphatic tissue (4). Some patients develop atrophic autoimmune thyroiditis, which is characterized by thyroid gland fibrosis, reduction of blood perfusion, and severe damage of thyroid tissue resulting in rapid progression of severe hypothyroidism. This atrophic thyroiditis is considered the form of autoimmune thyroid disease associated with lower levels of anti-thyroid antibodies in comparison to the goitrous form of AIT. It is typical that the majority of patients with atrophic AIT are diagnosed at the phase of advanced overt hypothyroidism (5). The diagnosis of AIT in those patients is usually delayed, probably because of the lack of goiter. In such children, some characteristic changes in the clinical and biochemical picture can be observed. Although the symptoms of hypothyroidism seem to be well-known, they are still often overlooked. Stereotypically, a child with hypothyroidism is an obese, short, slow, and sleepy patient with a goiter. Nonetheless, particular symptoms usually have a specific pattern: in children with hypothyroidism, there is an observed redistribution of subcutaneous tissue due to myxedema rather than simple obesity; growth arrest is more characteristic than absolute short stature; and weight gain is observed with normal or even reduced food intake. Some patients also present non-specific symptoms like fainting and headaches. In laboratory tests, the most characteristic abnormalities are lipid disorders (6) and anemia (7, 8), but in some cases liver (9) and kidney (10) function could also be impaired.

The aim of our study was to analyze the clinical and biochemical profiles of children with severe primary hypothyroidism due to autoimmune thyroiditis.

## Materials and Methods

The study was retrospective. The medical records of 354 patients with AIT diagnosed between 2009 and 2019 in our institution were reviewed. Only patients with severe hypothyroidism (SH) were enrolled in the study. The criteria of inclusion were thyroid-stimulating hormone (TSH) value above 100 μIU/mL associated with free thyroxine (fT4) concentration below reference values and the presence of antithyroid antibodies: anti-thyroid peroxidase antibodies (anti-TPO ab) and/or anti-thyroglobulin antibodies (anti-Tg ab). Twenty-six patients (17 girls, nine boys) aged 10.26 ± 3.3 years met these criteria.

Serum concentrations of TSH, fT4, free triiodothyronine (fT3), anti-Tg ab, anti-TPO ab, aspartate aminotransferase (AST), alanine aminotransferase (ALT), creatinine, urea, fasting glucose, total cholesterol (total-C), low-density lipoprotein cholesterol (LDL-C), high-density lipoprotein cholesterol (HDL-C), triglycerides (TG), and complete blood cell count (CBC) were analyzed. TSH receptor antibodies (TSHR ab) were measured in six out of 26 patients. Bone age (BA) using the Greulich and Pyle method (11) was evaluated in 14 out of 26 patients. An ultrasound of the thyroid gland was performed in each patient using 7.5–11 MHz linear transducer. In patients with specific indications, magnetic resonance imaging of pituitary gland was also performed.

The study was approved by the Bioethics Committee at the Medical University of Warsaw.

### Biochemical Analysis

The serum concentrations of TSH, fT4, fT3, anti-Tg ab, and anti-TPO ab were measured by the immunofluorescence method using the Architect i1000SR Analyzer (Abbott Diagnostics, Abbott Park, Illinois, USA). The anti-TSHR ab levels were measured by electrochemiluminescence immunoassay (ECLIA) with the Cobas e801 Analyzer (Diagnostics Roche, Basel, Switzerland). ALT and AST activity, creatinine, and urea concentrations were measured by the dry chemistry method using Vitros 5600 Analyzer (Ortho Clinical Diagnostics, Raritan, New Jersey, USA). The glomerular filtration rate (GFR) was calculated using creatinine level, age, sex, and height by Bedside Schwartz method (12).

Fasting glucose was determined in blood serum using Vitros 5600 Analyzer (Ortho Clinical Diagnostics, Raritan, New Jersey, USA). The lipid profile parameters were determined using Vitros 5600 Analyzer (Ortho Clinical Diagnostics, Raritan, New Jersey, USA). CBC was measured in blood collected in EDTA samples using Sysmex-XN-1000i hematological analyzer (Sysmex Europe, Norderstedt, Germany): red blood cell (RBC) count, hemoglobin (Hgb), mean corpuscular volume (MCV), mean corpuscular hemoglobin (MCH), mean corpuscular hemoglobin concentration (MCHC), total white blood cell (WBC) count, and platelet (PLT) count. Blood samples were obtained from patients after overnight fasting.

Reference values of analyzed laboratory parameters are shown in Table 1.

**Table 1:** Average values of all evaluated biochemical parameters in SH children.

**Parameter**	**Results**	**Reference limits**
**Thyroid**		
TSH (μIU/ml)	454.3 (310.4 – 899.0)	0.58 – 3.59
fT4 (ng/dl)	0.39 (0.39 – 0.43)	0.84 – 1.47
fT3 (pg/ml)	1.48 ± 0.66	2.33 – 4.35
Tg ab (IU/l)	934.0 (197.4 – 1902.7)	<4.1
TPO ab (IU/l)	195.4 (38.3 – 647.1)	<5.6
**Liver**		
ALT (U/l)	49.0 (25.0 – 108.0)	10 – 30
AST (U/l)	73.0 (41.0 – 98.0)	10 – 40
Fasting glucose (mg/dl)	81.7 ± 8.0	70 – 99
**Kidneys**		
Creatinine (mg/dl)	0.7 (0.6 – 0.8)	0.2 – 0.7
Urea (mg/dl)	28.1 ± 7.0	15 – 36.4
**Lipids**		
total-C (mg/dl)	255.0 (214.0 – 343.0)	<170
LDL-C (mg/dl)	170.4 (145.0 – 244.0)	<110
HDL-C (mg/dl)	57.6 ± 22.9	>45
TG (mg/dl)	109.5 (67.0 – 173.0)	<90
**CBC**		
RBC (×10^6^/μl)	4.1 ± 0.6	4.7 – 6.1
Hgb (g/dl)	12.0 ± 1.5	14 – 18
MCV (fl)	87.8 ± 5.3	78 – 95
MCH (pg)	29.2 ± 1.7	26 – 32
MCHC (g/dl)	33.4 ± 1.0	31 – 35
WBC (×10^3^/μl)	6.7 ± 2.0	4 – 10
PLT (×10^3^/μl)	256.8 ± 64.5	150 – 400

*Data are presented as mean ± standard deviation or median with interquartile range as appropriate*.

### Statistical Analysis

Statistical analysis was performed using Statistica 13.1. Data was checked by the Shapiro-Wilk normality test. Results were reported as means ± standard deviation (SD), median and interquartile ranges (IR), or as percentages, as appropriate. Correlations between variables were evaluated using the Spearman's correlation analysis for non-normally distributed data and the Pearson's correlation test for normally distributed data. A *p* < 0.05 was considered significant.

## Results

### Clinical Symptoms in SH Children

**Figure 1:**
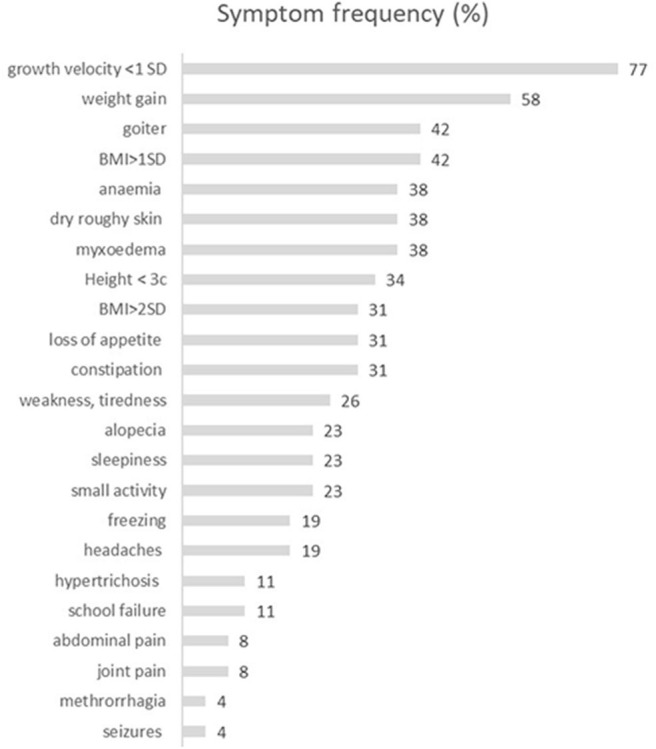
The characteristics of the frequency of symptoms reported by patients with severe hypothyroidism. BMI, body mass index [kg/m^2^].

The mean age at diagnosis was 10.26 (range 3.0–14.85), with girls' preponderance 1.8:1 (17:9). Growth arrest was the most frequent symptom (77%), whereas absolute short stature (height <3rd percentile for Polish population) was present only in 38%. The second most frequent symptom was weight gain, reported in 58% of patients; simultaneously, absolute obesity was found in 38% (*n* = 10) of patients (Table 2). Only 11 out of 26 patients (42%) had a goiter confirmed by ultrasound volume evaluation (Figure 1). The rarest symptoms were headaches, reported by four (15%) patients, and among them, seizures in one patient (4%). In those four patients, the CNS imaging was performed and anterior pituitary hypertrophy of variable grade was found without focal changes. Three patients had substantial hypertrichosis on the whole skin area, which disappeared when euthyroidism was achieved again. The characteristics of all clinical symptoms and findings in the SH children are presented in Figure 1. We also analyzed the diagnostic delay in SH children. The mean putative time from the occurrence of the first symptoms to the moment of diagnosis ranged from 6 months to 3 years.

**Table 2:** The auxological characteristics and hormonal and antibodies profile in patients with severe hypothyroidism.

**No**	**Age (years)**	**Sex**	**TSH (mIU/l)**	**fT4 (ng/dl)**	**fT3 (pg/ml)**	**Anti-TPO (nl <5.6)**	**Anti-Tg (nl <4.1)**	**Anti-TSHR (positive >1.75IU/L)**	**Goiter**	**Bone age (years)**	**Height SDS**	**BMI (kg/m^**2**^)**	**BMI (SDS)**	**Puberty Tanner scale**
1	3	F	>1,000	0.41	1.4	49.0	17.0		Yes	2	−3.99	19.1	2.42	B1P1
2	4.59	F	279.64	0.43	2.37	9340.9	61.2		No		0.16	28.9	8.50	B1P1
3	5.34	F	859.74	<0.4	0.99	140.0	2630.6		No	2.5	−1.8	19.6	2.39	B1P1
4	5.67	M	534.45	<0.4	0.99	4899.6	143.6		No	4	−2.13	14.9	−0.54	G1P1
5	6.75	F	332.4	0.4	1.6	2001.0	1400.0		Yes	6	−1.29	18.3	1.21	B1P1
6	8.5^twin^	F	>1,000	0.15	0.92	187.0	36.8	0.8	No	3.5	−3.5	24.5	3.18	B1P1
7	8.5^twin^	F	359.0	0.48	2.74	383.6	416.9	0.8	No	5	−2.4	21.7	2.04	B1P1
8	8.59	F	171.22	0.5	2.52	7.7	250.9		Yes		1.06	30.4	4.59	B1P1
9	9.08	F	>1,000	<0.4	0.99	65.5	784.8	1.8	No	8	−1.53	22.2	1.75	B1P1
10	9.67	M	278.37	0.45	2.5	2756.7	283.5		Yes		−0.34	20.3	1.04	G1P1
11	10	F	561.43	<0.4	0.99	124.5	26.6	0.33	No		0.18	22.2	1.75	B1P1
12	10	F	316.97	<0.4	1.49	2668.9	440.8		No	8	−1.8	24.3	2.47	B1P1
13	10.17	F	355.78	<0.4	1.31	1804.4	15.7		No		−0.36	23.4	1.96	B1P1
14	10.5	M	134.59	0.48	1.86	541.4	225.2		No	9	1.66	24.8	2.45	G1P1
15	10.58	M	362.13	0.51	1.49	476.1	29.8		No		−1.3	23.5	1.65	G1P2
16	11.68	M	962.85	<0.4	1.11	1185.8	554.8	0.8	No	8.5	2.17	18.6	0.15	G1P1
17	11.75	F	899.0	0.04	0.25	1589.7	1761.0	0.77	Yes		−1.06	24.3	1.92	B2P2
18	12	F	>1,000	<0.4	1.49	653.0	69.7		Yes		−0.21	18.0	−0.36	B2P2
19	12.09	M	613.39	<0.4	0.99	1723.3	21.9		No	11	−2.06	25.9	2.08	G2P2
20	12.17	M	447.21	<0.4	1.08	2806.2	739.4		Yes		0.06	22.5	1.19	G2P2
21	13.42	F	133.94	0.41	2.18	233.2	158.0		Yes		−1.46	17.1	0.88	B3P3
22	13.84	F	461.48	<0.4	1.1	1584.7	3712.0		Yes	12.5	−3.05	25.7	1.86	B3P3
23	14.67	M	310.4	<0.4	0.99	207.7	39.8		No		−0.22	25.8	2.17	G3P3
24	14.67	F	561.98	0.36	0.98	873.3	45.8		Yes	12	−3.6	19.1	−0.37	B3P4
25	14.67	F	100.28	0.49	1.93	994.6	165.5		Yes		0.61	19.1	−0.38	B4P4
26	14.85	M	>1,000	0.22	0.74	88.8	15.5		no	12.5	−2.1	21.5	1.04	G2P3

*TSH, thyroid stimulating hormone at presentation; fT4, free thyroxine value at presentation; fT3, free triiodothyronine value at presentation; anti-TPO, anti-thyroxine peroxidase antibodies and anti-Tg, antithyroglobulin antibodies (normal values in brackets); anti-TSHR, anti-TSH receptor antibodies; Height SDS, height values in SD standardized for age and sex; BMI, body mass index; BMI SDS, BMI values in SD standardized for age and sex; B, breast development; P, pubic hair development. Patients No. 6 and 7. were monozygotic twins*.

### Laboratory Findings

The evaluation of CBC revealed that 38% (*n* = 10) of patients had reduced RBC, 42% (*n* = 11) had decreased Hb value, and MCV in the majority of our patients was normal or slightly increased, with only one child having low MCV value.

Average values of laboratory results in all SH children are presented in Table 1. Parameters of lipid profile and kidney and liver function are shown in Table 3.

**Table 3:** Liver and kidney function tests in children with severe hypothyroidism.

**No**	**Age (years)**	**TSH (mIU/L)**	**ALT (U/L)**	**AST (U/L)**	**Creatynine (mg/dl)**	**Urea (mg/dl)**	**GFR (ml/min/1.73 m^**2**^)**	**Lipid profile**			
								**Total-C (mg/dl)**	**HDL-C (mg/dl)**	**LDL-C (mg/dl)**	**TG (mg/dl)**
1	3	>1,000	62	90	0.6		56.4	183	27	134.8	106
2	4.6	279.64	25	39				221			
3	5.3	859.74	114	133	0.7	33	60.8	613	88	494.4	153
4	5.7	534.45	49	85	0.7	27	61.7	235	58	146	153
5	6.7	332.4	52	58	0.6	30	77.8	286	85	189.6	57
6	8.5	>1,000	25	49	0.5	28	93.3	230	36	145	119
7	8.5	359.0	19	43	0.5	39	98.3	208	50	148	48
8	8.6	171.22	21	29	0.6	23	94.2	214			
9	9.1	>1,000	139	98				289	86	192.8	51
10	9.7	278.37	151	86	0.6	21	94.2	214	30		477
11	10	561.43	44	92	0.8	27	72.8	411	51	348	81
12	10	316.97	35	46	0.5	27	61.4				
13	10.2	355.78	48	55	0.9	27	63.8	400	27	195.4	296
14	10.5	134.59						189	62	114.6	62
15	10.6	362.13			0.8	28	69.2	281			224
16	11.7	962.85	108	144	1.1	38	50.7	253	71	156	58
17	11.75	899.0	59	101.7	1.1	41	53.7	343	23	244	383
18	12	>1,000	26	41				283			162
19	12.1	613.39	113	128	1.1	41	52.4	375	84	254	113
20	12.2	447.21	97	98	0.8	19	79.5	366	57	272	184
21	13.4	133.96	24	40	0.6	14	106.0	208	78	114.8	76
22	13.8	461.48	16	25	0.4		146.6	255	80	160.6	72
23	14.7	310.4	82	159	1.5	25	45.9				
24	14.7	561.98	108	73	0.8		85.9				
25	14.7	100.28	14	16	0.8	31	86.2	164	44	99.4	103
26	14.9	>1,000									

### Relations of TSH, fT4, and fT3 Levels With Other Biochemical Parameters and CBC

In SH children, TSH levels were significantly positively correlated with AST (*R* = 0.46, *p* = 0.026) and urea levels (*R* = 0.50, *p* = 0.043). Negative correlations were found between TSH and RBC (*R* = −0.75, *p* < 0.0001) and between TSH and Hgb (*R* = −0.74, *p* < 0.0001). TSH concentrations and MCV were positively correlated (*R* = 0.46, *p* = 0.019). Surprisingly, lipid profile turned out to not be significantly correlated with TSH value in SH children, although lipid parameters were highly increased (Tables 1, 3).

Free T4 levels were strongly negatively correlated with total-C (*R* = −0.66, *p* = 0.001) and LDL-C (*R* = −0.61, *p* = 0.009). Positive correlations were found between fT4 and certain CBC parameters such as RBC (*R* = 0.45, *p* = 0.023), Hgb (*R* = 0.45, *p* = 0.024), MCHC (*R* = 0.49, *p* = 0.015), and PLT (*R* = 0.46, *p* = 0.021). Free T4 concentration was negatively correlated with MCV (*R* = −0.57, *p* = 0.003). Unfortunately, the evaluation of fT4 associations was limited, because in most patients at diagnosis we received the laboratory result of fT4 concentration as a value of “ <0.4 ng/dl” (Table 2), so for statistical calculations we determined the value arbitrarily as 0.39 ng/dl.

We found a statistically significant negative correlation between fT3 levels and ALT (*R* = 0.46, *p* = 0.046) and AST (*R* = −0.68, *p* = 0.001). A strong negative correlation was found between fT3 and total-C (R = −0.67, *p* = 0.002). Free T3 also correlated with CBC parameters: positively with RBC (*R* = 0.53, *p* < 0.05) and Hgb (*R* = 0.47, *p* = 0.031), and negatively with MCV (*R* = −0.58, *p* < 0.05) and MCH (*R* = −0.45, *p* < 0.05).

Bone age was delayed more than 1 year in nine out of 14 children in whom it was determined. The median difference between chronological age and BA was 0.4 years, with the range from 0 to 5 years. We found a significant negative correlation between TSH value and absolute BA (*R* = −0.55, *p* = 0.040), but no correlation between TSH and degree of BA delay (delta in years) (*p* = 0.08).

No relationships between anti-Tg ab or anti-TPO ab and any evaluated parameter were found in our study. Antibodies against TSHR were evaluated only in six out of 26 SH patients at the presentation and in one of them the value was positive: 1.8 IU/l (by the cut-off 1.75 IU/l). None of our patients had thyroid ophthalmopathy.

## Discussion

### Clinical Symptoms

Growth arrest in children is the most sensitive symptom of thyroid hormone deficiency because of the thyroid's strong impact on growth hormone synthesis and action. Nevertheless, in our group of patients, only 77% reported decreased growth velocity as the main problem. We can easily explain this observation taking into consideration the age of our patients and their pubertal status. All patients in whom the growth retardation was not reported were at the age of 13–14 years and had almost reached their final height and completed their pubertal development. Additionally, comparing the number of children with growth inhibition and absolute short stature, we can conclude that growth arrest, rather than height below the third percentile, is the most sensitive symptom of hypothyroidism. Similarly, comparing the frequency of weight gain (58%) and BMI >2SD (38%) in our group of SH patients, it can be concluded that it is not obesity, but fast weight gain that is typical of hypothyroidism.

The next observation concerns goiter. It seems to be a strange finding that goiter occurence is so rare and not of very high volume in SH children under such a strong stimulation of extremely increased TSH (Table 2). In the literature, there is evidence that some patients with atrophic AIT could be positive for anti-TSHR ab (13, 14) that act as TSH-blocking factors. They are usually polyclonal and bind to the leucine-rich repeat region of the extracellular domain of the TSHR, similar to stimulating antibodies present in patients with Graves' disease (15, 16). Blocking antibodies cut off the signal pathway of TSHR, and this can cause atrophic changes in the thyroid gland. Nevertheless, in our group, the number of patients in whom anti-TSHR ab were determined was too small to make reliable conclusions. In our opinion, in hypothyroid patient without goiter, anti-TSHR antibodies should be the obvious element of laboratory workshop.

For the last 10 years, reports evaluating large groups of children with severe hypothyroidism have been scanty and most of them concern cases with some unusual manifestations. Cabrera et al. (17) reported a relatively large group of 62 children with severe primary hypothyroidism. In this group, eight (24%) patients at prepubertal age experienced pseudoprecocious puberty with such symptoms as thelarche and/or menarche in girls and isolated testicular enlargement in boys, which regressed during the thyroid hormone replacement. In de Vries et al., the group of 114 children and adolescents with autoimmune thyroiditis reported normal onset and duration of puberty; however, in their study, only 40 children were hypothyroid with TSH value between 11.8 and 236 mIU/l (18). In our study also, none of the patients presented symptoms of precocious puberty. The only abnormality related to puberty in our group was excessive menstrual bleeding in one girl at the age of 14.7 years. It is a typical symptom of hypothyroidism in menstruating girls, which is connected with impaired production of coagulation factors in the liver (19). Additionally, primary hypothyroidism is often associated with increased prolactin level, which can cause puberty arrest and oligomenorrhea or secondary amenorrhea. Khawaja et al. reported 16 patients younger than 20 with severe hypothyroidism (TSH >50 mIU/l) where two girls presented precocious puberty with precocious menarche and breast development with prepubertal response to gonadotropin-releasing hormone stimulation test (20).

Skin symptoms present in our patients, such as myxedema, dry, rough skin as well as loss of head hair are widely known as symptoms of hypothyroidism and are easily identified by patients and doctors. As an unusual skin symptom presented by SH children, we consider substantial hypertrichosis of individual intensity, which disappeared in the course of thyroid hormone treatment. Hypertrichosis accompanied by hair loss has been reported in hypothyroid patients (21). The question is why the same patient can suffer loss of head hair and, simultaneously, significant hypertrichosis on other skin areas. To explain this phenomenon, one has to take into account the fact that skin symptoms are dependent not only on thyroid hormone deficiency, but also on excessive TSH level. Thyroid hormone receptor Beta 1 has been proved to be expressed in the human hair follicle (22), but also TSHR messenger RNA (mRNA) and protein have been detected in human scalp hair follicles (23). Western blot and immunohistochemical analyses of skin specimens have confirmed the presence of TSHR protein in keratinocytes and fibroblasts (24). Moreover, it has been found that TSH treatment can induce the proliferation of cultured keratinocytes and fibroblasts (24). We can presume that the hypertrichosis observed in SH children could be dependent on prolonged TSH excess, whereas the head hair loss could rather result from thyroid hormones deficiency.

It is worthy to notice that in our group of SH children, at the moment of diagnosis none have the associated autoimmune comorbidities such as celiac disease, adrenal insufficiency, or diabetes mellitus, which is often observed in adult patients with Hashimoto's thyroiditis. Our data are concordant with observation of Ruggeri et al. (25), who reported that association between HT and other autoimmune diseases increases with age and occurs most frequently in adults.

### Laboratory Characteristics

It is widely known that anemia is associated with hypothyroidism (26). Its prevalence in adults has been reported at 14–43% in overt hypothyroidism (26, 27). The evaluation of vitamin B12, iron, and folic acid levels in hypothyroid patients with anemia has not revealed any significant differences (26, 27). In our group of SH children, anemia was present with similar frequency (38%), although one might have expected a higher prevalence in severe hypothyroidism. The positive correlation of TSH level and MCV in our study supports the hypothesis of Das et al. (28), which suggests that the basic background of anemia in hypothyroidism is a deficiency of erythropoietin dependent on thyroid hormone deficiency. Nevertheless, the differences in the values of Hgb, MCV, and RBC among our patients also suggest that the pathophysiology of anemia in hypothyroidism is much more complicated and must have various reasons including iron deficiency in some cases.

Hypothyroidism is closely associated with abnormal liver function resulting in atherogenic lipid profile and elevated aminotransferases. Liver function tests return to normal during thyroid hormone replacement (29). Nevertheless, increased prevalence of non-alcoholic fatty liver disease in hypothyroid adults with abnormal alanine aminotransferase according to the grade of hypothyroidism has been reported (30).

In our study, we found an increase of cholesterol and its fractions in SH patients, but we did not observe any evident correlations of lipid parameters with the TSH level. On the contrary, very clear associations were found between fT4 or fT3 concentrations and lipid profile. We suppose that perhaps extremely increased TSH values should be evaluated for statistical use in logarithmic scale, because the deviations of TSH values in our study were very high in comparison to deviations in lipids concentrations. In our group of SH children, aminotransferases were elevated in the majority of patients in whom they were measured (*n* = 23). We observed a preponderance of AST elevation: it was detected in 82% (*n* = 19) of children and ALT increased in 65% (*n* = 15) (Tables 1, 3). The value of TSH correlated positively only with AST concentration. Most authors suggest that more frequent AST than ALT elevation in hypothyroidism results from associated myopathy, not only the liver injury (30). On the other hand, other factors could also be involved in pathomechanism of the injury, including oxidative stress and decreased ceruloplasmin level, which is reported in hypothyroid patients (31, 32).

Myopathy and rhabdomyolysis have been reported as the factors partially responsible for the increase of creatinine, which is released from muscles (33). Serum creatinine elevation has been observed in adults as well as in children with hypothyroidism (34). This change can normalize during the treatment with thyroxine; however, in a long-standing study by Elgadi et al. (35), the authors suggest that renal impairment induced by hypothyroidism in children is not as benign as has been previously considered. In their study, GFR did not normalize completely even after 5 years of thyroxine therapy in some patients (35).

In our group of SH children, creatinine concentration was elevated in four patients, but calculated GFR was decreased in 11 children and was determined only in 15 out of 26 patients. It shows that this is not considered a routinely examined parameter in hypothyroid patients. The pathomechanism of renal impairment in hypothyroidism is not fully understood. It is suggested that thyroid hormones have an influence on muscle function, circulating volume, and cardiac function, and also have a direct effect on the kidney (36).

### Imaging

#### Bone Age

In general experience, BA in hypothyroidism is delayed proportionally to the grade and duration of thyroid hormones deficiency. In our group, the BA was determined in patients with significant height deficiency (below third percentile) and we had a unique opportunity to observe monozygotic twin girls at the age of 8.5 years with severe hypothyroidism with a different delay of BA (3 and 5 years, respectively) (Table 2). They had similar thyroid hormone deficiency, but the duration of hypothyroidism was longer in the girl with greater BA delay.

#### Thyroid Ultrasonography

In ultrasound scans, thyroid glands in our patients were hypoechogenic, 11 out of 26 patients (42%) had goiter, and a small thyroid was found in three children (11.5%). Thyroid blood perfusion was increased in 53.8%, and reduced in 11.5%. Nodules or focal changes were present only in two out of 26 SH children. They were of benign character.

#### Pituitary MRI

An interesting finding was pituitary hyperplasia found in four SH children suffering from headaches. One of those four children also had seizures. The incidence of pituitary hyperplasia secondary to hypothyroidism is unknown and probably underestimated. Pituitary MRI in patients with hypothyroidism is recommended only when some suggestive symptoms are present, i.e., vision disturbances, seizures, or severe headaches. A recent review of literature by Cao et al. (37) reported only 17 pediatric cases published from 1980 to 2017 (retrieved from Pubmed). On the other hand, Shukla et al. (38) published a review of pituitary hyperplasia in adult patients with hypothyroidism, which suggests that it is an underestimated problem and the frequency of pituitary hyperplasia has not been clearly identified. Khawaja et al. (20) reported that pituitary enlargement is observed in 70% of patients with TSH value >50 mIU/L and in 84% of patients with TSH higher than 100 mIU/L. Additionally the authors suggested that in younger patients, the frequency of pituitary enlargement can be even higher, but in their study there were only 16 patients below 20 years of life (20).

In our group of SH children, the MRI was performed only in four out of 26 children and the indications for this examination were headaches or neurological symptoms, not hypothyroidism. In these four children, everyone had an enlarged anterior pituitary and the function of tropic hormones secreted in pituitary was not impaired (Supplementary Table 1).

In SH patients, a deep deficiency of thyroid hormones causes excessive over-secretion of thyrotropin-releasing hormone (TRH) resulting in hypersecretion of TSH and prolactin. Increased prolactin and enlarged pituitary can suggest false pituitary adenoma, moreover the overlapping clinical symptoms such as growth arrest and weight gain associated with pituitary-suprasellar tumors could be differentiated with other childhood suprasellar tumors, i.e., craniopharyngioma. The differential diagnostics can be easy considering thyroid tests results because patients with craniopharyngioma typically present secondary and not primary hypothyroidism. More difficult could be the differentiation from prolactinoma. Another problem is not to overlook TSH-secreting adenomas, which were sporadically reported in patients with severe longstanding hypothyroidism due to the possible autonomisation of thyrotrophs (39, 40). Histology studies in humans revealed some characteristic changes of pituitary cells known as “thyroidectomy cells” (41) and additionally, it is suggested that thyrotroph hyperplasia is attributed to a loss of inhibitory feedback of the hypothalamo-pituitary axis (42). In concert to these findings, it could be presumed that patients without goiter positive for blocking anti-TSHR ab might be particularly predisposed to pituitary enlargement because of the interference of these antibodies with the ultrashort loop (TSH-pituitary) (43), which could additionally enhance the pituitary enlargement.

## Final Conclusions

In children with severe hypothyroidism, the most sensitive symptoms are growth arrest and weight gain, despite the fact that, in some children, the auxological parameters at presentation could be within normal values for the population. The specific biochemical profile is closely correlated with severity of thyroid hormone deficiency and involves mostly erythropoiesis, liver function, and kidney function. Pituitary enlargement should be considered in each child with severe hypothyroidism. It is necessary to conduct prospective studies evaluating the actual frequency of anti-TSHR antibodies and pituitary enlargement in children with extremely high TSH, especially without goiters.

## Data Availability Statement

All datasets presented in this study are included in the article/Supplementary Material.

## Ethics Statement

The studies involving human participants were reviewed and approved by Bioethics Committee at the Medical University of Warsaw. Written informed consent to participate in this study was provided by the participants' legal guardian/next of kin.

## Author Contributions

AK designed the study, wrote, and supervised the manuscript. EW-S performed statistical analysis and wrote the manuscript. DL and MR recorded data of the patients, wrote the manuscript, and collected the literature data. All authors have read and approved the manuscript.

## Conflict of Interest

The authors declare that the research was conducted in the absence of any commercial or financial relationships that could be construed as a potential conflict of interest.
