# Supplementary material for: Clinical and Biochemical Characteristics of Severe Hypothyroidism Due to Autoimmune Thyroiditis in Children
Source: Front Endocrinol (Lausanne). 2020 Jul 8;11:364. doi: 10.3389/fendo.2020.00364 (PMC7360718; doi:10.3389/fendo.2020.00364)
Supplement: Supplementary file 1 [file Table_1.docx]

Supplementary Table 1.

Characteristics of SH patients with documented pituitary hyperplasia

| No | sex | Age  [years] | TSH  [mIU/L] | fT4  [ng/dl] | Pituitary size  (ap x trans x cc)  [mm] | Cortisol [µg/dl] | ACTH [pg/ml] | IGF1  [ng/ml] | Prolactin  [ng/ml] |
| --- | --- | --- | --- | --- | --- | --- | --- | --- | --- |
| 6 | F | 8.5 | >1000 | 0.15 | 10x21x13 | 7.2 | 14.8 | 123.3 | 64 |
| 7 | F | 8.5 | 359.5 | 0.48 | 16x8x8 | 8.8 | 16.8 | 93.6 | 20.4 |
| 16 | M | 11.7 | 962.85 | <0.4 | 20x11x16 | 9.9 | 19.1 | 112 | 31 |
| 26 | M | 14.85 | >1000 | 0.22 | 21x12x15 | 8.7 | - | 120.3 | 34 |
